# Association Between Circulating Zinc and Risk for Childhood Asthma and Wheezing: A Meta-analysis on 21 Articles and 2205 Children

**DOI:** 10.1007/s12011-023-03690-4

**Published:** 2023-05-05

**Authors:** Mei Xue, Qiong Wang, Bo Pang, Xiaoqian Zhang, Yicheng Zhang, Xiangling Deng, Zhixin Zhang, Wenquan Niu

**Affiliations:** 1 Graduate School, Beijing University of Chinese Medicine, Beijing, China; 2 Department of Pediatrics, China-Japan Friendship Hospital, Beijing, China; 3 International Medical Services, China-Japan Friendship Hospital, Beijing, China; 4 Institute of Clinical Medical Sciences, China-Japan Friendship Hospital, Beijing, China

**Keywords:** Asthma, Wheezing, Children, Meta-analysis, Circulating zinc

## Abstract

Asthma is one of the most frequent chronic diseases in children, and growing focus is placed on the exploration of attributable risk factors. Currently, no consensus has been reached on the implication of circulating zinc in the development of asthma. We aimed to conduct a meta-analysis to examine the association between circulating zinc and risk for childhood asthma and wheezing. We searched PubMed, Web of Science, EMBASE, and Google Scholar from inception until December 1, 2022. All procedures were performed independently and in duplicate. Random-effects model was adopted to derive standardized mean difference (SMD) and 95% confidence interval (95% CI). Statistical analyses were completed using the STATA software. Twenty-one articles and 2205 children were meta-analyzed. Overall, there was a statistically significant association between circulating zinc and risk for childhood asthma and wheezing (SMD: −0.38; 95% CI: −0.60 to −0.17; *I*^2^=82.6%, *p*<0.001), without evidence of publication bias as revealed by Begg’s (*p*=0.608) and Egger (*p*=0.408) tests. Subgroup analyses showed that children with asthma or wheezing in Middle Eastern countries had significantly lower circulating zinc levels than controls (SMD: −0.42; 95% CI: −0.69 to −0.14; *p*<0.001; *I*^2^=87.1%). Additionally, average circulating zinc levels in asthma children were 0.41 μg/dl lower than that in controls, and the difference was statistically significant (SMD: −0.41; 95% CI: −0.65 to −0.16; *p*<0.001; *I*^2^=83.7%). By contrast, children with wheezing were 0.20 μg/dl lower than that in controls, and no between-group difference was noted (SMD=-0.20; 95% CI: −0.58 to 0.17; *p*=0.072; *I*^2^=69.1%). Our findings indicated that circulating zinc was associated with a significant risk for childhood asthma and its related symptom wheezing.

**Supplementary Information:**

The online version contains supplementary material available at 10.1007/s12011-023-03690-4.

## Introduction

Asthma is one of the most frequent chronic diseases affecting children globally. Currently, the prevalence of asthma is rising [1], and it poses a heavy burden on individuals and public health systems. Global statistics have shown that approximately 340 million persons are experiencing asthma and related symptoms (such as wheezing), causing nearly 1000 deaths per annum [2]. With the increasing prevalence and morbidity resulting from asthma and wheezing, growing focus is on the exploration of attributable risk factors, the prevention of mortality, and the promotion of pulmonary health. Thus far, circulating zinc is widely evaluated, yet its association with asthma and wheezing remains poorly understood [3–5].

Zinc, a critical micronutrient, is related to many life structures and physiological processes of the human body, especially in terms of anti-oxidative stress and anti-inflammatory effects [6]. Importantly, zinc is reported to regulate innate and adaptive immune systems and maintain immune tolerance. It is widely accepted that zinc deficiency can cause a panel of symptoms, such as depressed immune function, growth retardation, frequent infections, and mental disturbances [7, 8]. Globally, zinc deficiency may affect up to 2 billion people [9]. However, there is no consensus on the association between zinc and asthma incidence. Some studies supported the contributory role of zinc in asthma prevention [10–19], whereas others failed to support this claim [20–26]. The association between zinc and asthma hence remains an open question.

The implication of zinc in the development of asthma is biologically plausible. First, zinc deficiency may disturb the balance between type 1 and type 2 T helpers, resulting in inflammation and eosinophilia [27, 28]. Second, zinc deficiency may be associated with the production of IgE, which increases the risk of asthma [29, 30]. Third, zinc is of powerful antioxidant activity in the lungs, which may be responsible for the imbalance between oxidation and antioxidant, ultimately reducing antioxidant function and increasing the risk of asthma [31, 32]. Altogether, it is reasonable to speculate that circulating zinc is a promising marker in susceptibility to asthma. Previously, Ghaffari and colleagues [33] pooled the results of 15 studies, and failed to support the association of serum zinc with asthma in children. With accumulating studies afterwards, there is a need to reinterrogate this association.

To yield more information, we conducted an updated meta-analysis with the aim of exploiting the association between circulating zinc and risk for childhood asthma and wheezing, as well as seeking possible causes for between-study heterogeneity.

## Methods

This meta-analysis complied with the Preferred Reporting Items for Systematic Review and Meta-analysis (PRISMA) [34], and the *PRISMA checklist* is shown in *Data Sheet* *1*.

### Search Strategy

Literature search in the PubMed, Web of Science databases, EMBASE (Excerpt Medica Database), and Google Scholar was commenced from November 1, 2022 to December 1, 2022. The searches were conducted to identify all published studies that reported data on mean differences and standard deviations of circulating zinc in patients with asthma and/or wheezing and healthy controls. Key terms for literature searching were generated based on the MeSH database: (zinc OR Zn OR trace element OR element, trace OR trace mineral OR mineral, trace OR microelement OR nutrient element OR anti-oxidant OR antioxidant OR anti-oxidant OR endogenous antioxidant OR antioxidants, endogenous OR antioxidant, endogenous OR activity, antioxidant OR antioxidant OR anti-oxidant* OR micronutrients OR micronutrient) [Title/Abstract] AND (asthma OR bronchial asthma OR asthma, bronchial OR asthmatic OR asthma* OR wheeze OR wheez* OR atopy OR atopic disease OR airway hyperresponsiveness OR respiratory hypersensitivity OR airway inflammation OR intermittent airway obstruction OR paroxysmal dyspnea) [Title/Abstract] AND (child OR children OR child* OR adolescent OR adolesc* OR teenage OR teen* OR youth OR young OR pediatr*) [Title/Abstract] AND (longitudinal study OR prospective study) [Title/Abstract]. Literature search was conducted by two investigators (M.X. and Q.W.).

### Selected Criteria

Studies were included if they met the following criteria simultaneously: [1] children younger than 18 years old; [2] cases are diagnosed as asthma or asthma-related symptom wheezing; [3] prospective design; [4] available circulating zinc levels; [5] extractable data to infer levels of zinc in serum or plasma presented as mean and standard deviation (or data were available to calculate them).

Studies were excluded if either one of the following criteria was met: [1] lack of control groups; [2] use of infant umbilical cord blood; [3] in form of case reports/series, reviews, or conference abstract.

Two investigators (M.X. and Q.W.) independently reviewed all retrieved articles and carefully assessed preliminary eligibility. All titles and abstracts were thoroughly checked. Full text was read when necessary.

### Data Extraction

Data were extracted on the basis of following items: surname of first author, publication year, country where study was conducted, ethnicity, continent, the feature of case (asthma or wheezing), diagnosis of asthma or wheezing, source of controls, blood sample (serum, plasma, or blood), matching of potential confounders, techniques for measuring zinc levels, fasting (fasting, non-fasting or not available), the respective numbers of boys and girls in the case and control groups, sample size, average age for case and control groups, as well as mean and standard deviation for zinc levels, data units, the Zn/Cu ratio in cases and the Zn/Cu ratio in controls. Zinc levels were converted to the uniform unit μg/dL. All the information was independently entered into an Excel sheet by two researchers (M.X. and Q.W.). Disagreement was adjudicated by a third researcher (W.N.).

### Statistical Analysis

The STATA software v14.1 was used to analyze data. The association of circulating zinc levels with asthma and related symptoms was quantified using standardized mean difference (SMD) and 95% confidence interval (CI). Heterogeneity test, known as homogeneity test, is an integral element of meta-analysis, and its purpose is to test whether the statistics of multiple similar studies are heterogeneous. The inconsistency index (*I*^2^) was used to quantify the magnitude of statistical heterogeneity. The larger the *I*^2^ value is, the greater the heterogeneity is. When *I*^2^ is lower than 50%, heterogeneity is acceptable [35]. In this meta-analysis, random-effects model was executed to calculate effect-size estimates. Generally speaking, fixed-effects models are often used in studies where differences between results arise solely from sampling error, that is, there is no statistical heterogeneity. Moreover, in the absence of statistical heterogeneity, the results of the fixed-effects model and the random-effects model are very similar [36].

As there are various sources of heterogeneity, cumulative analyses were conducted to assess the impact of the first publication on subsequent publications and the evolution of the accumulated estimates over time. In addition, influential analyses were conducted to assess the effect of any individual study on overall effect-size estimates by omitting one study at a time, and its main role is to evaluate the stability of the meta-analysis model.

Begg’s funnel plot and Egger’s regression asymmetry test were adopted to judge the probability of publication bias. If funnel shape is asymmetrically inverted, it might suggest a correlation between pooled estimate and study size (publication bias or small study bias). The trim-and-fill method was used to estimate the number of potentially missing studies. Significant publication bias is recorded if the probability of Egger test is less than 10%. Clinical and methodological heterogeneity across studies was assessed by means of meta-regression analyses and subgroup analyses (according to ethnicity, continent, diagnosis of asthma, source of controls, blood sample (serum, plasma, or blood), matched condition, and fasting status (fasting, non-fasting or not available)).

## Results

### Eligible Studies

**Fig. 1:**
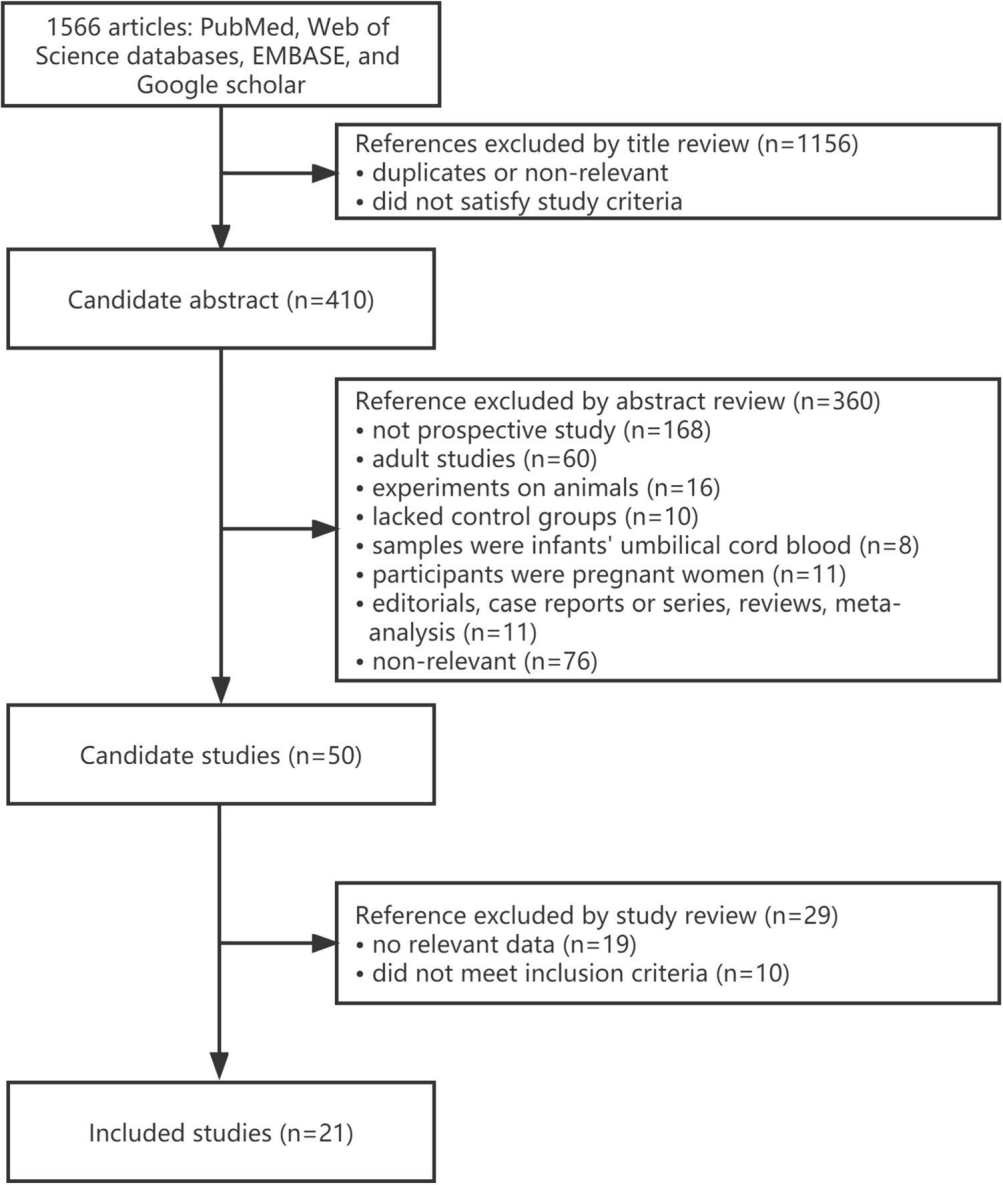
The Preferred Reporting Items for Systematic Reviews and Meta-analyses (PRISMA) flow diagram of study selection with specific reasons for exclusion

A total of 1566 potentially eligible articles were identified, and of them 21 articles involving 2205 children throughout rigorous screening were qualified for this meta-analysis(10-–26, 37–40). The detailed selection procedure is shown in Fig. 1.

### Study Characteristics

The baseline information for all included studies is shown in Table 1. All studies were published from the year 1987 to 2021. The studies were divided into three groups based on ethnicity, including Middle Eastern [10, 12–16, 18, 21, 22, 24–26, 37–39], African [11, 23, 40], and Caucasian [17, 19, 20]. Circulating zinc was detected by atomic absorption spectrometry in 12 studies [11, 13, 15–18, 22–24, 26, 39, 40], calorimetric method in 3 studies [14, 21, 37], inductively coupled plasma mass spectrometry in 2 studies [10, 38], laser-induced breakdown spectroscopy in 1 study [12]; the measurement method of 3 studies was not reported [19, 20, 25].

**Table 1:** **The baseline characteristics of all involved studies in this meta-analysis**

First author	Year	Country	Ethnicity	Continent	Feature of cases	**Diagnosis**		Source of controls	Blood sample	Matched	Technique	Fasting	
Yalçın	2021	Turkey	Middle Eastern	Asia	Asthma	GINA		Population	NA	Yes	Non-AAS	NA	
Kuti	2020	Nigeria	African	Africa	Asthma	GINA		Population	Serum	Yes	AAS	NA	
Alsharnoubi	2020	Egypt	Middle Eastern	Africa	Asthma	GINA		Hospital	NA	NA	Non-AAS	NA	
Andino	2019	America	Caucasian	America	Asthma	Doctor		Hospital	Serum	Yes	NA	NA	
Elevli	2018	Turkey	Middle Eastern	Asia	Asthma	Doctor		Hospital	Serum	Yes	Non-AAS	NA	
AbdulWahab	2018	Qatar	Middle Eastern	Asia	Asthma	GINA		Hospital	Serum	Yes	AAS	Non-fasting	
Wagdy	2017	Egypt	Middle Eastern	Africa	Asthma	Doctor		Hospital	Serum	Yes	Non-AAS	NA	
Uysalol	2014	Turkey	Middle Eastern	Asia	Wheezing	ERS/ATS		Hospital	Serum	Yes	AAS	Fasting	
Oluwole	2014	Nigeria	African	Africa	Asthma	ISAAC		Population	Plasma	Yes	AAS	NA	
Khanbabaee	2014	Iran	Middle Eastern	Asia	Asthma	GINA		Population	Serum	Yes	Non-AAS	NA	
Bilan	2014	Iran	Middle Eastern	Asia	Asthma	Doctor		Hospital	Serum	Yes	AAS	Fasting	
Ghaffari	2013	Iran	Middle Eastern	Asia	Asthma	Doctor		Hospital	Serum	NA	AAS	Fasting	
Kakarash	2012	Iraq	Middle Eastern	Asia	Asthma	Doctor		Hospital	Serum	Yes	AAS	NA	
Razi	2011	Turkey	Middle Eastern	Asia	Wheezing	Doctor		Population	Serum	Yes	Non-AAS	NA	
Behmanesh	2010	Iran	Middle Eastern	Asia	Asthma	Doctor		Hospital	Serum	Yes	NA	NA	
Kocyigit	2004	Turkey	Middle Eastern	Asia	Asthma	Doctor		Population	Plasma	NA	AAS	Fasting	
Ermis	2004	Turkey	Middle Eastern	Asia	Asthma	ERS/ATS		Hospital	Serum	NA	AAS	NA	
Malvy	1993	France	Caucasian	Europe	Asthma	GINA		Hospital	Plasma	Yes	AAS	NA	
el-Kholy	1990	Egypt	Middle Eastern	Africa	Asthma	Doctor		Hospital	Serum	Yes	AAS	NA	
Di Toro	1987	Italy	Caucasian	Europe	Asthma	Doctor		Population	Serum	Yes	NA	Fasting	
Akinkugbe	1987	Nigeria	African	Africa	Asthma	Doctor		Hospital	Plasma	NA	AAS	NA	
Boys		Girls		Sample size		Age (years)		Zinc levels (mean)		Zinc levels (SD)		Zn/Cu ratio	
Cases	Controls	Cases	Controls	Cases	Controls	Cases	Controls	Cases	Controls	Cases	Controls	Cases	Controls
9	14	8	12	17	26	7.7	7.8	586.00	586.00	116.00	116.00	4.99	5.89
52	50	28	30	80	80	7.2	7.3	71.00	84.20	30.30	31.70	NA	NA
30	12	10	28	40	40	8	8	2.96	4.62	0.81	1.28	10.20	6.51
NA	NA	NA	NA	24	24	13	14	7.65	9.10	2.55	3.03	NA	NA
25	35	25	35	50	70	8.7	9.6	81.17	87.62	26.04	32.76	NA	NA
34	32	6	8	40	40	10.9	10.9	830.04	850.32	117.74	99.42	NA	NA
22	10	16	7	38	17	3.3	3.2	249.63	148.47	93.97	60.92	1.36	1.18
46	43	27	32	73	75	1.5	1.5	0.07	0.07	0.01	0.02	0.54	0.60
27	24	10	6	37	30	13.5	13.5	10060.00	10630.00	2280.00	2470.00	2.00	1.99
55	55	45	45	100	100	5.4	5.3	70.50	80.90	22.60	16.90	NA	NA
36	32	14	18	50	50	5.7	5.1	20.12	25.20	10.14	8.95	0.20	0.33
77	78	98	87	175	165	10.3	11.4	83.08	85.06	44.96	20.35	0.72	0.87
27	27	23	23	50	50	6.5	6.5	70.02	84.04	7.78	10.08	NA	NA
56	68	44	48	100	116	2.3	2.5	69400.00	78900.00	16500.00	29900.00	0.63	0.75
NA	NA	NA	NA	80	80	6.1	5.3	93930.00	97180.00	25580.00	23590.00	NA	NA
24	19	20	13	44	32	8.2	7.3	76.57	77.55	16.72	10.64	0.55	0.58
19	13	22	17	41	30	7.6	7.6	70.60	78.30	8.30	9.20	0.49	0.60
6	12	NA	NA	6	12	9	9	0.75	0.92	0.14	0.18	NA	NA
NA	NA	NA	NA	22	20	7	7	70.30	88.40	13.20	11.00	0.88	1.30
11	12	11	7	22	19	6.9	7.7	1.06	1.06	0.11	0.23	0.69	0.79
NA	NA	NA	NA	20	20	5.5	5.4	89.20	79.00	21.90	61.60	2.01	1.02

*GINA* Global Initiative for Asthma, *ERS/ATS* European Respiratory Society/American Thoracic Society, *ISAAC* International Study of Asthma and Allergies in Childhood, *AAS* atomic absorption spectrometry, *NA* not available

### Overall Analyses

**Fig. 2:**
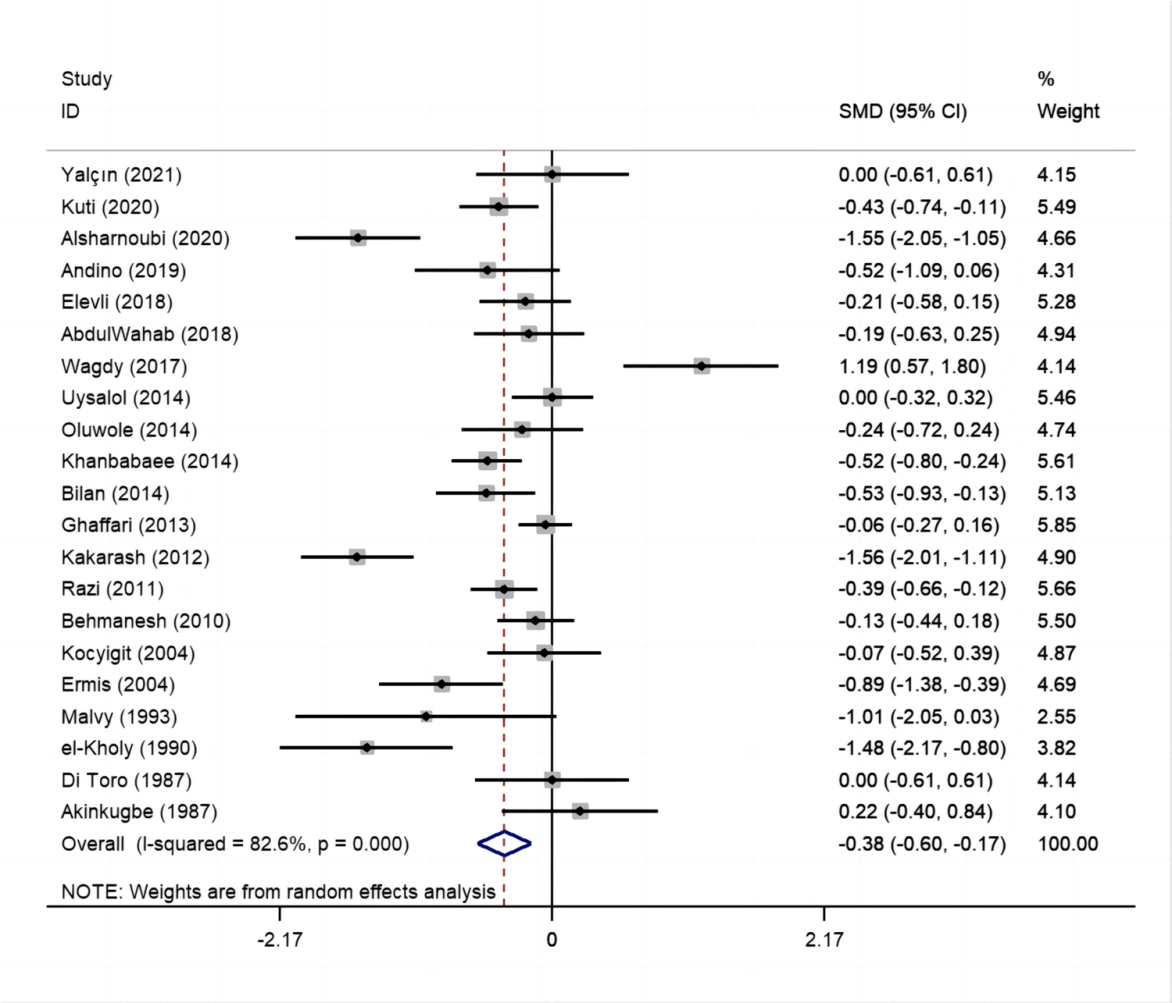
Forest plot of circulating zinc and risk for childhood asthma and its related symptom wheezing. Abbreviations: SMD, standardized mean differences; 95% CI, 95% confidence interval

Via pooling the results of all eligible studies together, there was a statistically significant correlation between circulating zinc levels and risk for childhood asthma and its related symptom wheezing (SMD: −0.38; 95% CI: −0.60 to −0.17; *I*^2^=82.6%, *p*<0.001) (Fig. 2).

### Cumulative and Influential Analyses

**Fig. 3:**
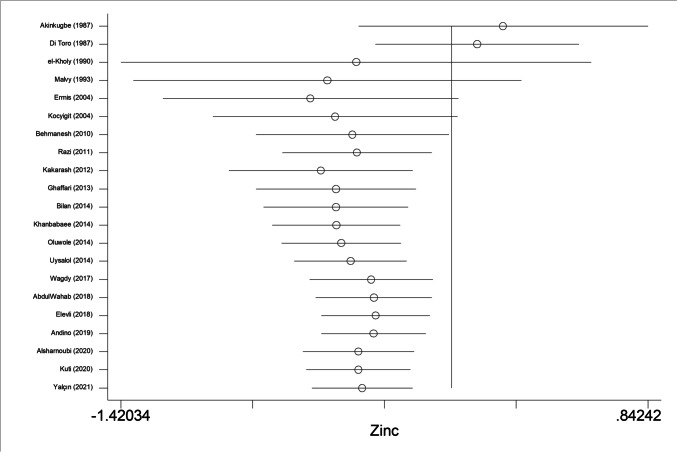
Cumulative analyses of 21 studies for the association between circulating zinc and risk for childhood asthma and its related symptom wheezing

**Fig. 4:**
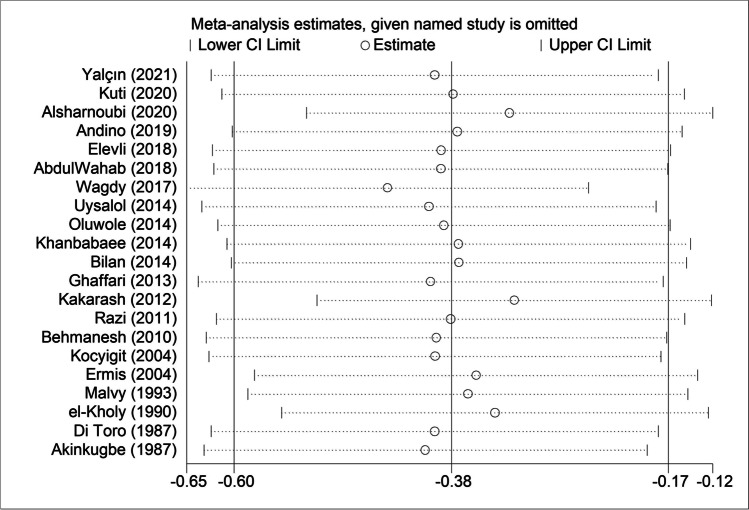
Influential analyses of 21 studies for the association between circulating zinc and risk for childhood asthma and its related symptom wheezing

In sensitivity analyses, the first study carried out in 1987 by Akinkugbe and colleagues had no significant impact on subsequent studies (Fig. 3). In influential analyses, the point estimates after deleting any study were all within 95% CI of total effect size, revealing no significant impact of any single studies on overall effect sizes for childhood asthma and wheezing (Fig. 4).

### Publication Bias

**Fig. 5:**
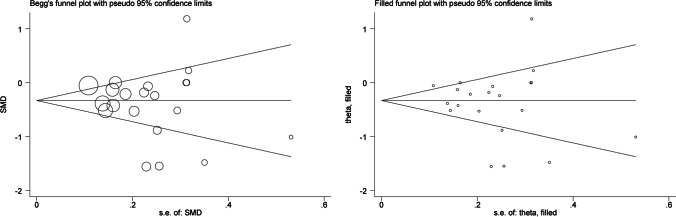
Begg’s and filled funnel plots of 21 studies for the association between circulating zinc and risk for childhood asthma and its related symptom wheezing

Figure 5 shows that the funnel plot was symmetric, and publication bias was not significant (Begg’s test *p*=0.608; Egger’s test *p*=0.408). Also, as reflected by filled funnel plots, there was no missing study.

### Regression Analyses

Meta-regression analyses were performed because of the significant heterogeneity observed between studies, by separately regressing average age of cases, average age of controls, percentage of boys in cases, percentage of boys in controls, Zn/Cu ratio in cases and Zn/Cu ratio in controls (Table 2). There was no observable significance for above factors (*p*>0.05).

**Table 2:** Meta-regression analyses of potential factors.

Factors	*N*	Std.Err.	t	*p*	95% CI
Average age of cases	21	0.04	-0.79	0.43	-0.12, 0.05
Average age of controls	21	0.04	-0.72	0.47	-0.11,0.05
Percentage of boys in cases	17	0.01	-0.90	0.37	-0.03,0.01
Percentage of boys in controls	17	0.01	0.90	0.38	-0.01,0.02
Zn/Cu ratio in cases	13	0.06	-1.47	0.14	-0.23, 0.03
Zn/Cu ratio in controls	13	0.09	-1.17	0.24	-0.30, 0.07

*N* number of included studies; *Std.Err* standard error; *t* t statistic, the coefficient divided by its standard error; *p* an independent variable would be significant (<0.05) or not significant (≥0.05) in the model; *CI* confidence interval

### Subgroup Analyses

It is widely acknowledged that clinical and methodological diversity across studies often leads to statistical heterogeneity. To address this diversity, subgroup analyses were conducted accordingly (Table 3). By ethnicity, the association between circulating zinc and risk for childhood asthma and its related symptom wheezing was stronger in Middle Eastern than in other ethnicities. Additionally, children with asthma or wheezing in Middle Eastern had significantly lower circulating zinc levels than controls (SMD: −0.42; 95% CI: −0.69 to −0.14; *p*<0.001; *I*^2^=87.1%). By feature of case, average circulating zinc levels in asthma patients were 0.41 μg/dl lower than that in controls, and the difference was statistically significant (SMD: −0.41; 95% CI: −0.65 to −0.16; *p*<0.001; *I*^2^=83.7%). By contrast, children with wheezing were 0.20 μg/dl lower than that in controls (SMD: −0.20; 95% CI: −0.58 to 0.17; *p* =0.072; *I*^2^=69.1%), and the difference was nonsignificant.

**Table 3:** Subgroup analyses of circulating zinc with risk of asthma and wheezing

Subgroups	*N*	SMD	95% CI	*p*	*I*^2^
By ethnicity					
African	3	−0.23	(−0.57, 0.11)	0.188	40.2%
Caucasian	3	−0.41	(−0.91, 0.10)	0.213	35.4%
Middle Eastern	15	−0.42	(−0.69, −0.14)	<0.001	87.1%
By the feature of case					
Asthma	18	−0.41	(−0.65, −0.16)	<0.001	83.7%
Wheezing	3	−0.20	(−0.58, 0.17)	0.072	69.1%
Diagnosis of asthma					
Doctor	12	−0.30	(−0.61, 0.01)	<0.001	85.7%
GINA	6	−0.58	(−0.97, −0.19)	<0.001	77.7%
Non-GINA	3	−0.35	(−0.87, 0.16)	0.013	77.0%
By the source of controls					
Hospital	14	−0.46	(−0.79, −0.13)	<0.001	88.1%
Population	7	−0.34	(−0.48, −0.20)	0.436	0.0%
By blood sample					
NA	2	−0.79	(−2.30, 0.73)	<0.001	93.2%
Plasma	4	−0.15	(−0.50, 0.20)	0.237	29.1%
Serum	15	−0.37	(−0.61, −0.17)	<0.001	83.6%
By detection technology					
AAS	12	−0.48	(−0.77, −0.19)	<0.001	82.4%
NA	3	−0.18	(−0.43, 0.07)	0.418	0.0%
Non-AAS	6	−0.28	(−0.78, 0.23)	<0.001	89.8%
By fasting status					
Fasting	5	−0.12	(−0.30, 0.06)	0.274	22.0%
NA	15	−0.49	(−0.79, −0.20)	<0.001	85.3%
Non-fasting	1	−0.19	(−0.63, 0.25)	NA	NA
By matched					
NA	5	−0.47	(−1.05, 0.12)	<0.001	89.5%
Yes	16	−0.36	(−0.60, −0.12)	<0.001	80.5%

*N* number of included studies, *SMD* standardized mean difference, *I*^2^ percentage of total variation across studies, *CI* confidence interval, *GINA* Global Initiative for Asthma, *Non-GINA* refers to ERS/ATS and ISAAC, *AAS* atomic absorption spectrometry, *NA* not available

## Discussion

Via a comprehensive analysis of 21 articles and 2205 children, our findings indicated that circulating zinc was associated with the significant risk for childhood asthma and its related symptom wheezing, which reinforced our speculation that circulating zinc is a promising marker in susceptibility to asthma. To our knowledge, this is thus far the most comprehensive meta-analysis that has investigated the association between circulating zinc and risk for childhood asthma and its related symptom wheezing in the literature.

Zinc deficiency is one of the most important micronutrient deficiencies globally. In recent years, much interest has been aroused by the possibility that zinc deficiency may increase the incidence of morbidity and mortality from asthma and other respiratory diseases. Many studies suggested that zinc deficiency may be related to the IgE production, which can increase the risk of asthma, that is, asthmatic children tend to have higher IgE levels than individuals without asthma [43]. Besides, the initiation of asthma may be through oxidative stress or inflammation. Zinc intake was inversely associated with incidence of asthma, which could decrease hyper-responsiveness [44]. Understanding the relationship between zinc status and risk for asthma or wheezing is therefore of critical importance to generate evidence-based recommendations. There may be a potential for zinc interventions to reduce children’s susceptibility to asthma or wheezing, which might contribute to a more profound protection by zinc against asthma or wheezing.

Currently, hair and nail zinc is not recognized as substitutes for body zinc; therefore, this meta-analysis was aimed only at investigating the association between circulating zinc and asthma or wheezing in children. In 2021, Ghaffari and colleagues [10] meta-analyzed the association between serum zinc levels and children’s asthma by pooling the results of 15 articles, and they found no statistical significance in overall analyses. Based on the accumulation of literature and comprehensive statistical analysis, we come to the exact opposite conclusion. More importantly, using a relatively large number of eligible articles, we further explored potential causes of between-study heterogeneity by conducting a large panel of subgroup analyses. Irrespective of the differences between the study by Ghaffari and colleagues and the present meta-analysis, we need to heed the danger of lower circulating zinc for the early detection and prevention of childhood asthma.

Key findings of our meta-analysis suggested that lower circulating zinc levels were associated with higher risk for asthma or wheezing in children. In addition, we noted that low circulating zinc was more pronounced in asthmatic children than in wheezing children, but even if it was not so prominent, zinc levels in wheezing patients were still low, reminding us that once a child is diagnosed with wheezing, it is necessary to pay attention to circulating zinc levels early and replenish it as early as necessary.

It is intriguing to note that the association between circulating zinc and the risk for asthma/wheezing varies across ethnicities, with the strongest association in Middle Eastern. We have postulated that dietary phytates, present in cereal grains and legumes, interfere with zinc absorption and contribute to zinc deficiency. This mechanism is seen in populations with diets high in grains and low in meats, such as areas of the Middle East [41]. In fact, zinc deficiency was first described in adolescent males in Iran and Egypt [42, 43]. In addition, the potential racial disparity in the association between zinc and asthma may be partially explained by exposures and lifestyles (such as genetic predisposition, environmental pollution, viral infection and cigarette smoking) in Middle Eastern countries [44]. Besides, the progressive zinc deficiency and subsequently increased asthma were also thought to be due to socioeconomic factors and differences in access to medical care [30, 45, 46]. Therefore, it will perhaps open the door to appropriate management to reduce asthma burden, at least in part, achieving optimal asthma control and reducing the risk of asthma exacerbations and mortality in the Middle East region.

Our findings indicated no significant difference between genders upon the association between circulating zinc and asthma or wheezing, in agreement with those of Oluwole [23] and Razi [38]. In addition, complex interactions between zinc, iron and copper exist, although the exact molecular mechanisms behind these interactions remain elusive [47]. There is competition between them, especially when one mineral is too much, it can interfere with the absorption of the other. Dietary iron may inhibit the absorption and utilization of zinc, and the effect of bivalent iron is greater than that of trivalent iron [48]. Several transporters involved in iron, copper, and zinc metabolisms may be involved in the absorption of other essential trace elements [49, 50]. Further studies at molecular levels are needed to elucidate the interactions and greatly facilitate their uptake processes. In our meta-analysis, zinc/copper ratio showed no significant difference between case and control groups, which may be due to limited data.

Lower Zn-levels with the likely accompanied iron-deficiency in asthmatic patients that may further explain the results we obtained. First, both iron and zinc are key elements and modulators of immune cells. Zinc deficiency is strongly influenced by iron deficiency, both associated with the same food source and both inhibited by phytate [51]. And it is widely recognized that iron deficiency can affect immune activation and promote the production of IgE caused by zinc deficiency to a certain extent. The possible mechanism is to promote the entry of specific antigens into the body, stimulate T lymphocytes, and transmit them to B lymphocytes to synthesize specific IgE [51–53]. Another important type of cell in lung immunity is regulatory T cells. Iron/zinc deficiency leads to immune activation and may lead to a Th1/ Th17-based immune response and maturation of B cells targeting plasma cells [54], resulting in the release of multiple active mediators from these cells that cause smooth muscle contraction, increase mucus secretion, increase vascular permeability and inflammatory cell infiltration, and thereby promote inflammation [51]. Second, serum iron concentration is not only regulated by the immune system, but probably affects the lung function of the body [55–58]. Lung cells must receive adequate amounts of iron to meet metabolic demands and ensure lung function and survival [59]. At the same time, lung cells must avoid excess iron, oxidative stress and the resultant damage that may impair lung function [59]. In addition, prolonged immune activation will lead to functional iron deficiency that, over time, will develop anemia of chronic diseases [55], in association with the occurrence of lung diseases [57, 60, 61].

Strengths of our meta-analysis include the most comprehensive literature summary to date, the comprehensive exploration of heterogeneity and the pooled SMD by using a random-effects model. Nevertheless, we are aware that this meta-analysis may have some limitations. First, all involved studies were cross-sectional or case-control in design, precluding further comments on the causality between circulating zinc and childhood asthma/wheezing. Second, we cannot completely ignore the likelihood of missing studies that remained unpublished, despite the fact that funnel plots and statistical tests showed a low likelihood of publication bias. Third, this is an updated meta-analysis involving sufficient sample sizes for overall analyses, yet within some subgroups such as by ethnicity the sample sizes might not be sufficient in African and Caucasian. Fourth, decreased serum zinc levels might lead to an overestimation of zinc-deficiency during asthma. So, it is worth carrying out more clinical research to value, foster, and commit to shed more light on the differences in zinc levels between children with previously diagnosed asthma but without recent attack and normal children, as well as extensively explore dynamic changes in zinc levels in asthma remission, mild asthma, moderate asthma, and severe asthma.

Taken together, our findings indicated that circulating zinc was associated with the significant risk for childhood asthma and its related symptom wheezing. For practical reasons, collective action should be taken to ensure children have access to timely intervention, and we also agree that further investigations are urgently needed to elucidate and confirm our results.

### Supplementary Information

ESM 1: PRISMA 2020 Checklist (DOCX 26 kb)
